# The rising tide of opioid use and abuse: the role of the anesthesiologist

**DOI:** 10.1186/s13741-018-0097-4

**Published:** 2018-07-03

**Authors:** Elena J. Koepke, Erin L. Manning, Timothy E. Miller, Arun Ganesh, David G. A. Williams, Michael W. Manning

**Affiliations:** 1 Division of General, Vascular and Transplant Anesthesiology, Department of Anesthesiology, Duke University, Box 3094, 2301 Erwin Road, Durham, NC 27710 USA; 2 Division of Regional Anesthesiology, Department of Anesthesiology, Duke University, Durham, USA; 3 Division of Pain, Department of Anesthesiology, Duke University, Durham, USA

**Keywords:** Perioperative medicine, Multimodal analgesia, Opioid-free anesthesia, Opioid-reduced anesthesia, Opioid epidemic, Enhanced recovery pathways

## Abstract

Opioid use has risen dramatically in the past three decades. In the USA, opioid overdose has become a leading cause of unintentional death, surpassing motor vehicle accidents. A patient’s first exposure to opioids may be during the perioperative period, a time where anesthesiologists have a significant role in pain management. Almost all patients in the USA receive opioids during a surgical encounter. Opioids have many undesirable side effects, including potential for misuse, or opioid use disorder. Anesthesiologists and surgeons employ several methods to decrease unnecessary opioid use, opioid-related adverse events, and side effects in the perioperative period. Multimodal analgesia, enhanced recovery pathways, and regional anesthesia are key tools as we work towards optimal opioid stewardship and the ideal of effective analgesia without undesirable sequelae.

## Background

Opioids are an effective form of analgesia for acute pain but, with over-prescription, have become detrimental to public health in the USA. In the 1990s, a patient advocacy movement focused on pain control was born, and physician, patient advocacy, and professional societies began to promote aggressive treatment of both acute and chronic pain with opioids. The movement was propagated by many well-intentioned clinicians; however, the pharmaceutical industry had a hand in its creation and distribution. Although not a comprehensive summation of the causes of the opioid epidemic, the following events illustrate that a multitude of parties were complicit in its evolution:

1. In 1996, the American Pain Society declared that pain should be assessed “with the same zeal (as) other vital signs….”
2. In 1999, the VA Hospital System declared pain as “the fifth vital sign.”
3. JCAHO, the hospital credentialing agency, followed suit and educated health professionals to perform a consistent assessment and treatment of pain, as well as mandated the documentation of pain scores (Alam 2016).
4. During this time, the Federation of State Medical Boards also released a pain control policy that encouraged the use of opioids in the treatment of chronic pain.

Incidentally, the educational media promoted by JCAHO and the FSMB policy on chronic pain were funded by the pharmaceutical industry, or written by members who were on the payroll of a prominent pharmaceutical company. As a result, use and prescriptions for opioids began to multiply. The misleading claims and treatment guidelines were not brought to light until the USA was in the midst of an opioid epidemic.

**Fig. 1:**
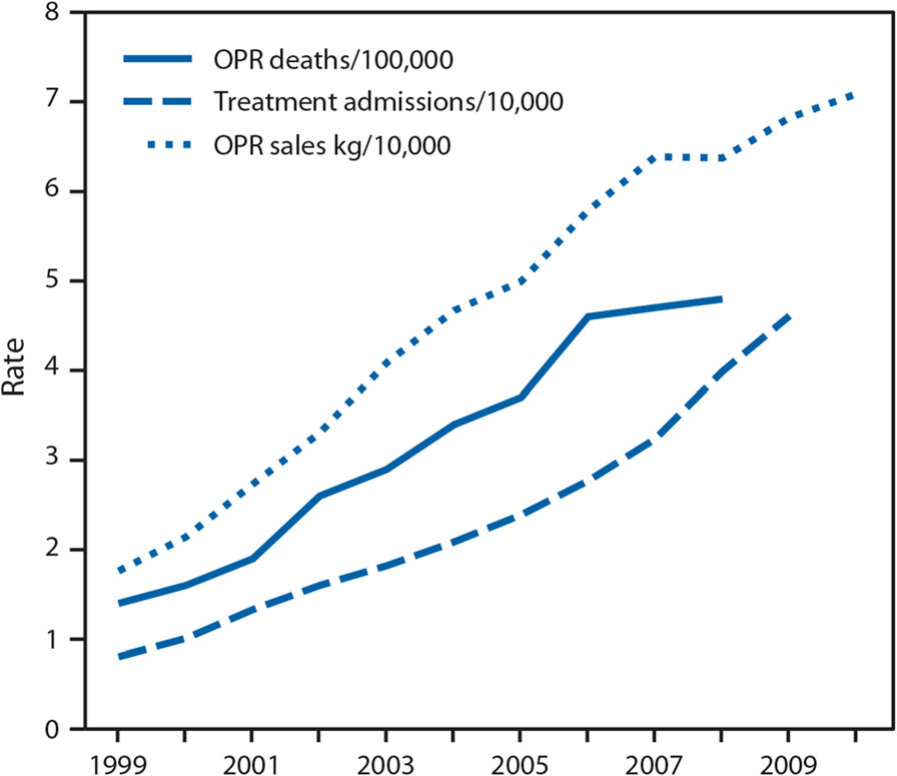
Rates of opioid pain reliever overdose death, opioid pain relief treatment admissions, and kilograms of opioid pain relievers sold—United States, 1999–2010. Age-adjusted rates per 100,000 population for opioid pain reliever (OPR) deaths, crude rates per 10,000 population for OPR abuse treatment admissions, and crude rates per 10,000 population for kilograms of OPR sold. From Centers for Disease Control and Prevention (2011)

Since 1999, there has been a steady increase in the number of opioids sold and opioid-related deaths (Manchikanti et al. 2012) (Fig. 1). In 2015, more than 40% of the world’s supply of thebaine, a main ingredient in hydrocodone and oxycodone, was consumed by the USA. Gram for gram, people in the USA consume more narcotic medication than other nations worldwide. By 2017, the number of opioid-related deaths has continued to increase (Hedegaard et al. 2017) and the US government has declared the opioid epidemic as a public health emergency.

In this review, we will briefly describe the opioid burden within the USA, with emphasis on the emerging evidence of a link between acute exposure to opioids and chronic opioid dependence. We will then highlight alternative approaches to the management of intraoperative and acute postoperative surgical pain which emphasize multimodal analgesics and thereby decrease the need for opioid use in the acute hospital setting.

### Perioperative use of opioids

In a recent survey of adult surgical patients, 75% had pain they rated as moderate/extreme during the immediate post-surgical period (Gan et al. 2014). Approximately 99% of all surgical patients in the USA receive opioids perioperatively at some point during their care (Kessler et al. 2013). Some argue, however, that opioids are used more frequently than needed due to increasing use of quality metrics that use pain control as a measure of quality of care (Zgierska et al. 2012). Despite the fact that opioids have significant side effects, they have historically been the primary treatment for post-surgical pain as they are very effective with a quick onset of action and no analgesic ceiling.

Opioid use, however, begets opioid use. Patients who received opioids for their primary pain therapy in the acute perioperative period are commonly to require increased doses to maintain the same analgesic effects (Chia et al. 1999). This is usually attributed to the development of “acute tolerance” to the analgesic effects of opioids, although this loss of analgesic efficacy may also be the result of opioid-induced hyperalgesia. Opioid-induced hyperalgesia has been defined as a state of nociceptive sensitization caused by exposure to opioids (Chu et al. 2008). Hyperalgesia and tolerance are very different pharmacologic phenomena that can lead to similar net increases in opioid dosing over time. This has been referred to as the *opioid paradox*: the more opioids used intraoperatively, the more opioids required postoperatively. The paradox has been measured in surgical patients up to 2 days postoperatively (Joly et al. 2005). Both acute opioid tolerance and opioid-induced hyperalgesia are the postulated mechanisms behind this phenomenon.

### Postoperative use of opioids and chronic opioid use

Using opioids to treat acute post-surgical pain was not originally believed to increase the risk of long-term opioid dependence or addiction in patients. An infamous letter published in a 1980’s *New England Journal of Medicine* quotes: “We conclude that despite widespread use of narcotic drugs in hospitals, the development of addiction is rare in medical patients with no history of addiction” (Porter and Jick 1980). However, recent literature suggests that some opioid-naïve patients became chronic users of opioids after surgery, chronic being defined as opioid use lasting > 90 days postoperatively. In a retrospective study of over 36,000 opioid-naïve patients undergoing elective surgery in the USA between 2013 and 2014, the incidence of chronic opioid use after surgery was ~ 6%. This did not differ between major and minor surgical procedures (Brummett et al. 2017).

Another study of opioid-naive patients undergoing short-stay surgery also found significant rates of chronic opioid dependence 1 year after surgery (Lee et al. 2017). Focusing on curative-intent cancer surgery in the USA from 2010 to 2014, Lee et al. found approximately 10% of patients were still using opioids 1 year after surgery.

The perioperative use of benzodiazepines, selective serotonin reuptake inhibitors (SSRIs), diabetes, younger age, lower income, higher intraoperative use, and the duration of acute postoperative opioid use have all been identified as factors which carry increased risk of prolonged postoperative opioid use (Clarke et al. 2014). A study by Shah et al. analyzed a one million patient sample of a large health insurance claim database which represented the US-insured population from 2005 to 2015. They found that postoperative prescribing patterns can effect the incidence of chronic opioid use disorder. Starting on the third postoperative day, every day of opioid therapy increases the risk of a patient becoming a chronic consumer of opioids—with the most dramatic increases after the fifth and 31st day of therapy. Cumulative prescription dose of ≥ 700 morphine milligram equivalents, initial prescription for a 10- or 30-day supply, or a second prescription or refill were also associated with an increased incidence of chronic opioid use disorder at 1 year postoperative. Moreover, the incidence of long-term usage is approximately 15% in patients whose first episode of consumption is for > 8 days, and this rises as high as 30% for patients whose initial acute opioid use lasts 31 days or more (Shah et al. 2017). This study may underestimate the true incidence of long-term usage as it focused on the American commercially insured population and did not include patients who paid for their prescriptions out of pocket or obtained opioids illicitly. Additionally, this study included all types of patients and was not limited to the perioperative population.

Given that a significant proportion of opioid-naïve patients entering surgery may ultimately become chronic opioid users, minimizing opioids during the perioperative period should be an important goal of anesthesiologists. Identifying the patients that are high risk for opioid misuse or opioid use disorder in perioperative clinics enables the perioperative team to construct a plan to minimize the length of time the patient is exposed to opioids inside and outside of the hospital.

### Acute side effects of perioperative opioids

Beyond increasing the risk of developing opioid use disorder, perioperative opioid consumption may produce undesirable side effects such as nausea/vomiting, constipation/ileus, pruritus, altered mental status, urinary retention, respiratory complications, and increased length of stay (Oderda et al. 2007). While many of these side effects are frustrating to patients in the immediate postoperative period, the most dangerous effects are those that affect the respiratory system.

Opioid-induced respiratory depression (ORD) is a significant cause of brain damage and death in the postoperative period. Cashman and Dolin reported their meta-analysis of almost 20,000 patients who underwent thoracic, abdominal, major gynecological, or major orthopedic surgery using a single postoperative analgesic technique with observation for adverse events for at least 24 h postoperatively (Cashman and Dolin 2004). The authors reported incidence of ORD varying from 0.1 to 37% based on their analysis, depending on the route of administration of the opioid, the type of opioid, the definition and method of monitoring ORD, and the prospective versus retrospective nature of the study. Lee et al. analyzed the Anesthesia Closed Claims Project Database and found that over 88% of ORD occurred within 24 h of the surgical procedure, and 13% of these occurred after the patients had been discharged from the PACU to the ward (Lee et al. 2015). The most common outcome for these ORD was death (55%) followed by recoverable injury (23%) and brain damage (22%).

### Postoperative prescribing: public health considerations

A small selection of studies has explored the quantity of opioids prescribed for various surgeries and the amount actually taken by patients following hospital discharge. Surgeons at Dartmouth found that 67% of prescribed opioids after common general surgeries were not taken by the patient (Hill et al. 2017). The quantity of pills prescribed was inconsistent and ranged from 0 to 120 pills.

Unused medications, including opioids, can subsequently be used for nonmedical purposes by the patient, his or her family, or others who may have access to improperly stored medications. In a survey of heroin users, approximately 75% reported that they initially started with a prescription opioid, often from another patient’s prescription (Jones 2013). In a study examining patients undergoing either cesarean section or thoracic surgery, investigators found that patients took fewer than five doses after discharge, and almost 80% of patients left their leftover pills in an unsecured location in their homes (Bartels et al. 2016; Rodgers et al. 2012). Almost none of the patients receiving opioids were given information on how to properly dispose these unused pills (Brummett et al. 2017).

Further research is needed to determine the appropriate amount of post-discharge opioids for each individual patient and procedure. We recommend that anesthesiologists assist their surgeon colleagues to establish institution-wide guidelines for postoperative opioid prescription. As anesthesiologists, we can provide verbal and written education on proper disposal techniques of opioids at perioperative clinic visits in an effort to decrease the distribution of leftover pills (Bicket et al. 2017). The perioperative anesthesia clinic visits are also an appropriate time to educate patients about the benefits of multimodal analgesia. A conversation should be held about our duty to ensure patient safety, and subsequently, we may avoid high doses of opioids with multimodal analgesia.

In addition, all physicians or providers who write for opioid prescriptions outside of the initial surgery encounter should be educated on the expected course length of an opioid prescription after a given surgery or injury. This will prevent primary care physicians from unnecessarily refilling prescriptions outside the normal time window and will also give them an idea as to when a patient is not healing properly or may be having a complication from surgery.

Despite the notoriety of the epidemic, it is still commonplace to use large amounts of opioids in the perioperative setting. Both anesthesiologists and surgeons are well-positioned to partner together to decrease the total amount of opioid used and prescribed in the preoperative, intraoperative, and postoperative settings. However, a multidisciplinary and multifaceted approach is needed to address the problem of the prescription opioid epidemic successfully. An important goal should be to start from the beginning and to ensure as many opioid-naïve patients remain opioid-naïve while having adequate pain control throughout their recovery.

## Contribution of multimodal analgesia

**Fig. 2:**
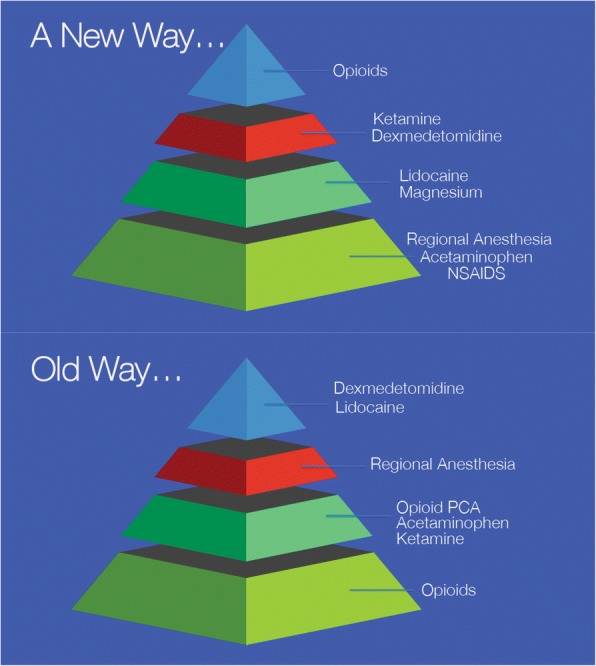
New paradigm in analgesia management. The old way of management of pain has relied on opioids as the foundation of pain control, with non-opioid adjuncts added if necessary due to patient condition. In the new way, management of pain begins with non-opioid-based techniques that are evidence based and demonstrated to decrease opioid use

Since the 1990s, a significant amount of research has gone into multimodal analgesia, as a methodology of pain control which utilizes non-opioid medications which target different pain receptors to better manage and control pain. The synergistic effect of the multiple medications also reduces the amount of opioid needed to control pain postoperatively, as opposed to using an opioid mono-therapy. Medications such as acetaminophen, gabapentin/pregabalin, ketamine, magnesium, dexamethasone, non-steroidal anti-inflammatory drugs (NSAIDs), duloxetine, and lidocaine are among those that have been studied as components of the multimodal approach (Fig. 2).

Acetaminophen has shown in systemic reviews (Macario and Royal 2011; Smith 2011) and clinical trials to reduce postoperative opioid use by as much as 30% at 4 h postoperatively (Aryaie et al. 2018; Memis et al. 2010; Saurabh et al. 2015; Sinatra et al. 2005). Although most trials concentrate on IV acetaminophen, a 2015 systematic review concluded that there is little evidence for using IV acetaminophen over oral acetaminophen in patients that are able to take medications by mouth perioperatively—which is useful when considering overall medication costs (Jibril et al. 2015).

There is conflicting evidence on whether or not the reduced opioid doses reduce opioid-related side effects or opioid-related adverse events in studies with concurrent opioid use (McNicol et al. 2011; Memis et al. 2010). A recent study using a nationwide database reported that in spine surgery patients, IV acetaminophen did not reduce inpatient use of opioids or opioid-related adverse events (Mörwald et al. 2018). The effectiveness of acetaminophen may be related to procedure and patient specifics. Additional trials are needed to make a definite conclusion about the ability of acetaminophen to reduce opioid-related adverse events.

Gabapentin and pregabalin are also commonly used in multimodal analgesia and have shown to reduce opioid consumption in the perioperative period. Randomized, placebo-controlled, and double-blind studies have shown that 1200 mg of perioperative gabapentin reduces postoperative morphine equivalents in various types of major procedures (Wick et al. 2017). A meta-analysis in 2007 showed that preoperative gabapentin doses ranging from 300 to 1200 mg decreased opioid use by 30 mg of morphine in the first 24 h post-procedure (Tiippana et al. 2007). Pregabalin has been shown in a meta-analysis to have similar effects on postoperative opioid reduction as well as reducing opioid-related side effects postoperatively (Engelman and Cateloy 2011; Mörwald et al. 2018).

NSAIDs are a very potent pain reliever and are often overlooked as an immediate perioperative medication due to concerns of side effects. When used in scheduled doses and in the correct patient population, they can be a powerful adjunct to any pain regimen. In their 2017 review on multimodal anesthesia, Wick et al. highlighted the findings that 600 mg of ibuprofen is efficacious as 15 mg of oxycodone hydrochloride (Wick et al. 2017). A long-held belief has been that NSAIDs, particularly ketorolac, increase the risk of surgical bleeding. However, Gobble et al. reported findings that ketorolac was not shown to increase incisional or GI bleeding in patients under 75 years old (Gobble et al. 2014). Recent meta-analyses in pediatric neurosurgery patients (Richardson et al. 2016) and plastic surgery patients (Stephens et al. 2015) both found no increased risk of postoperative bleeding.

A 2005 meta-analysis reported that NSAIDs in conjunction with opioid treatment significantly decreased pain scores at 24 h (Elia et al. 2005). It also reported that it decreased morphine consumption at 24 h by 50% with the COX-2 inhibitor rofecoxib and by 40% with other NSAIDs (Elia et al. 2005). The addition of NSAIDs or COX-2 inhibitor to an opioid pain regimen also decreased the incidence of nausea, vomiting, and sedation in both small and large surgeries. Although morphine consumption was significantly reduced, there was no reduction in opioid-adverse events (Elia et al. 2005). However, these trials reviewed within the meta-analysis were focused on efficacy of pain control. More focused studies are needed to show how a multimodal perioperative pain regimen can reduce opioid-related adverse events.

Perioperative administration of the alpha-2-agonists, clonidine and dexmedetomidine, has been shown to reduce postoperative opioid consumption by 4–15 mg of morphine equivalents, respectively, as well as reduced the pain intensity and nausea in patients (Blaudszun et al. 2012).

*N*-Methyl-d-aspartate (NMDA) antagonists are also commonly used medications in a multimodal pain regimen. Ketamine, a NMDA receptor antagonist, has long been known to decrease acute post-surgical pain and analgesic consumption. It is a very effective in helping to decrease tolerance to postoperative opiate use as well as being an effective analgesic ± opiates, non-steroidal anti-inflammatory medications, and acetaminophen (Katz 1993; McQuay 1992). Loftus et al. found that ketamine reduced opioid consumption in the postoperative period 0 to 48 h (Loftus et al. 2010). Moreover, these investigators found that opioid-dependent patients treated with ketamine had reduced pain up to 6 weeks after surgery compared to controls (Loftus et al. 2010).

More recently, Nielsen and colleagues reported that ketamine administered intraoperatively during spinal fusion surgery significantly decreased opioid use during the first 24 h postoperatively (Nielsen et al. 2017). In addition, these chronic pain patients with chronic opioid use receiving intraoperative ketamine had significantly less sedation and significantly improved pain scores at 6 months (Nielsen et al. 2017). This highlights ketamine’s long reaching effects at improving pain while achieving reduced use of opioids.

A 2015 meta-analysis by King et al. concluded that the low-affinity noncompetitive NMDA receptor antagonist dextromethorphan, a common ingredient in cough syrup, significantly decreased postoperative opioid consumption 24–48 h after surgery (King et al. 2016). This review of 14 trials and 848 patients found that preoperative dextromethorphan reduced morphine consumption by 10.5 mg postoperatively. It also decreased pain scores up to 24 h postoperatively.

Dexamethasone, long-used for inflammation and nausea/vomiting prophylaxis, is now gaining popularity as an adjunct in perioperative pain management. In 2011, De Oliveira et al. published a study that showed that doses of 0.11–0.2 mg/kg of dexamethasone were optimal for postoperative pain reduction and decreasing opioid consumption postoperatively (De Oliveira Jr. et al. 2011). This study was particularly important because, before this, less than 0.1 mg/kg dexamethasone was commonly given perioperatively for nausea/vomiting prophylaxis purposes. Additional studies continue to show the postoperative pain benefits of dexamethasone. A 2017 meta-analysis by Meng et al. found that dexamethasone significantly reduced opioid consumption for the entire 48 h after total joint arthroplasty as well as resulted in reduced pain scores (Meng and Li 2017).

In 2013, a systemic review concluded that magnesium significantly decreased early (3 h) postoperative and late (24 h) postoperative pain (De Oliveira et al. 2013). It also significantly reduced nausea and vomiting. Patients that received magnesium infusions intraoperatively and postoperatively had a greater reduction of pain score and greater reduction of morphine use than both control group and intraoperative magnesium use without accompanying postoperative administration (Bolcal et al. 2005; De Oliveira et al. 2013). Morphine use decreased on average more than 10 mg in the immediate postoperative period, although the total magnesium dose did not affect total postoperative opioid consumption. This may be due to the fact that magnesium was given intraoperatively and did not continue into the postoperative period. Despite the large decrease in postoperative opioid, magnesium did not decrease systemic adverse effects of morphine such as nausea or vomiting.

IV lidocaine infusions have been shown to reduce opioid consumption decrease postoperative pain, reduce hospital length of stay, and have earlier return of bowel function in open abdominal procedures (Vigneault et al. 2011). In some surgeries, this begs the question whether or not epidural infusions of local anesthetic are necessary. A study by Terwaki et al. reported that IV lidocaine infusion in major abdominal surgery was inferior to epidural analgesia with respect to opioid consumption. However, lidocaine was associated with improvements in several important aspects of recovery (Terkawi et al. 2016).

Duloxetine has been shown to significantly decrease morphine requirements after total abdominal hysterectomy, total knee arthroscopy, and spine surgery (Bedin et al. 2017). Studies differed on whether or not duloxetine reduced pain scores 48 h after surgery, but one could argue that opioid consumption is an objective measure of pain control as opposed to the subjective nature of pain scores. An animal-model trial in 2016 showed that it significantly reduced incisional pain in rats as well as allodynia and hyperalgesia (Wang et al. 2016). A review by Helander in 2017 on multimodal analgesia concluded that additional randomized controlled trials on duloxetine’s effect on postoperative reduction of opioids should be completed (Helander et al. 2017b).

## Regional anesthesia

The use of regional anesthesia is essentially a universally utilized component of any perioperative multimodal analgesia or enhanced recovery after surgery (ERAS) pathway (Grant et al. 2017). Both neuraxial and peripheral nerve blocks can be used concurrently with an opioid-free anesthetic. A patient may be able to remain opioid free from the first few hours after surgery and possibly for days depending on the type of block and surgery performed.

Epidural catheters play an important role in opioid reduction in ERAS pathways, as well as enhancing early recovery (Grant et al. 2017). One of the benefits cited for epidural use in open abdominal surgery ERAS pathways relates to the role epidural catheters play in reducing opioid-related adverse events (Grant et al. 2017). Specifically, adequate pain control through opioid-sparing analgesic techniques aids in the reduction of postoperative nausea and vomiting, ileus, somnolence, hyperalgesia, and postoperative delirium (Kim et al. 2016).

More recently, some of the focus on regional anesthesia/analgesia for a variety of abdominal surgical procedures (both as part of ERAS pathways and independently) has shifted to truncal fascial plane blocks, including transversus abdominal plane (TAP), rectus sheath, and quadratus lumborum blocks (Bashandy and Elkholy 2014; Garg et al. 2017; Helander et al. 2017a; Hutchins et al. 2015; Kadam 2013; Kim et al. 2016). While many of these fascial plane blocks have been performed as single injection with ropivacaine and bupivacaine, a desire to prolong the time to rescue with opioid analgesia (or to completely avoid opioids altogether) has led to use of both continuous indwelling regional catheters and liposomal bupivacaine (Hutchins et al. 2015; Mitra and Sinatra 2004; Stokes et al. 2017). Recent studies of transversus abdominis plane (TAP) blocks with liposomal bupivacaine for robotic-assisted hysterectomy and colorectal surgery showed lower pain scores, decreased postoperative opioid requirements in the first 72 and 36 h respectively (Hutchins et al. 2015; Stokes et al. 2017).

Additionally, regional anesthetic techniques are quite useful for opioid-tolerant patients who often have poorly controlled postoperative pain. In fact, it has been suggested that regional anesthesia/analgesia procedures be offered to opioid-tolerant patients whenever feasible (Mitra and Sinatra 2004). With the variety of regional techniques available and the flexibility of these techniques regarding different surgical procedures, regional anesthesia/analgesia uniquely positions the anesthesiologist to have a significant impact in the reduction of opioids in the perioperative period.

## Enhanced recovery pathways

Enhanced recovery pathways (ERPs) are multidisciplinary pathways aimed at reducing the stress of surgery, and thereby accelerating functional recovery. A key principle of ERPs is that anesthesia, surgery, and nursing team work together to decrease perioperative opioid use by implementing a multifaceted analgesia technique through all phases of perioperative care.

The goal is to deliver optimal analgesia. Optimal analgesia can be defined as “a technique that optimizes patient comfort and facilitates recovery of physical function, while minimizing the adverse effects of analgesics” (McEvoy et al. 2017).

To facilitate this goal, preoperative patient education is key. As pain is a complex and subjective biopsychosocial experience, optimal analgesia cannot be achieved without management of patient expectations and education regarding realistic goals of pain treatment. It is critical for the patients to understand that “pain-free” is not the primary goal of optimal analgesia.

The exact components of an enhanced recovery pathway will vary from institution to institution based on local factors, as well as the surgical and patient population. Importantly, there are many ways to achieve optimal analgesia that are efficacious. Each pathway should include a well-structured and planned multimodal approach from the preoperative period into the post-discharge recovery phase. It is also recommended that each pathway should have a “plan B” to allow for individual patient variation and a rescue analgesia pathway to troubleshoot breakthrough pain while continuing enhanced recovery principles and minimizing the negative effects of intravenous opioids.

High compliance with a variety of standardized non-opioid ERP bundles is strongly associated with a significant decrease in primary inpatient opioid consumption and improved outcomes in a variety of surgical procedures. Although multimodal analgesia routinely continues after hospital discharge, at present, most current care pathways in the USA do not include any guidelines on discharge opioid prescribing. With recent data showing that ERP implementation has not reduced the amount of opioid prescribed at discharge, this appears to be a missed opportunity and should be addressed as ERPs evolve (Brandal et al. 2017).

## Opioid-free anesthesia: present and future

Prior to the widespread adoption of opioids into anesthesia practice in the late 1960s, anesthesia (hypnosis, amnesia, and immobility) was obtained through the use of inhaled anesthetics and/or high-dose Pentothal. Unfortunately, this brought with it the complication of hemodynamic instability and suppression of hemodynamics requiring additional support.

With the introduction of opioids, came the concept of “balanced anesthesia” which was widely embraced for its lack of effect on the cardiovascular system. Opioids provide hemodynamic stability through suppressing the sympathetic system which is why they are preferred at induction. In addition, opioids are among the strongest analgesics, and analgesia then came to be an essential part of balanced anesthesia (Mulier 2017; Mulier 2016).

The introduction of opioids was important because hypnotics at the time were strong cardiovascular depressants. Many patients had undiagnosed or untreated cardiovascular coronary diseases, as understanding was limited at that time. So, by giving high doses of opioids allowed the anesthesiologists to reduce the doses of hypnotics and muscle relaxants (Bovill et al. 1984; Sebel and Bovil 1982). Today, hypnotics and neuromuscular blocking agents are much more stable and can be titrated to achieve a sufficient depth of hypnosis and muscle relaxation with minimal risk. And almost all patients today have been diagnosed and treated for their cardiovascular problems prior to presenting for elective surgery.

Now, there are drugs which can act to stabilize the sympathetic nervous system, including alpha-2 agonists such as clonidine and dexmedetomidine, local anesthetics given intravenously (lidocaine) (De Oliveira et al. 2012), and magnesium (De Oliveira et al. 2013). Drugs such as gamma-aminobutyric acid modulators such as gabapentin can decrease opioid requirements (Tiippana et al. 2007). Given alone, almost all of them will reduce the intraoperative opioid use when given in a sufficiently high dose, which may result in prolonged sedation. If, however, these are given together in a multimodal approach, it may be possible to avoid all intraoperative opioids and avoid excessive sedation. The advantage of such a multimodal approach is that postoperative opioids given as analgesics are then also dramatically reduced. It is worth noting that although primary care and pain physicians commonly combine many of these medications safely, we do not yet have literature on the safety of each of the different combinations among various age groups or procedures.

From an anesthetic perspective, one can perform an opioid-free anesthetic (OFA) while still providing satisfactory pain control. Jan Mulier and colleagues at Algemeen Ziekenhuis Sint-Jan Brugge-Oostende, Bruges, Belgium, have found that opioids can be completely eliminated from the anesthetics of patients undergoing bariatric surgery with improved outcomes especially in the area of obstructive sleep apnea (Mulier 2016). Mulier et al. performed a retrospective cohort of 5000 patients within their institution; a dramatic reduction in postoperative opioid use in the first 24 h (mean and standard deviation [SD] of morphine equivalents 21 mg and SD 0.99 versus 6 mg and SD 0.48) was reported, with fewer complications and a shorter length of stay (LOS). In this cohort, patients with obstructive sleep apnea (OSA) were not found to have more complications compared with non-OSA patients after OFA.

Similar reports have demonstrated that multimodal adjuncts can be utilized with similar outcomes to those of opioid use (Bakan et al. 2015; Steyaert and Lavand'homme 2018). We are left to wonder, why is not this approach adopted by the anesthesia community at large? OFA is something one has to learn. OFA is also a paradigm shift in thinking for many practitioners. At our own institution, our first OFA pathway was met with a healthy degree of skepticism; however, it is now become established for many minor-moderate pain procedures (Table 1) and has significantly reduced, if not altogether eliminated the requirement for opioids to control pain. Importantly, while opioids are not routinely given intraoperatively in any of our ERPs, they are always available postoperatively if needed. Our goal is not to leave patients in pain, but for many patients to complete their surgical journey with satisfactory pain relief without the need for opioids.

**Table 1:** Opioid-reducing enhanced recovery pathway. This is the protocol used at the authors’ institution for multimodal, opioid-reducing analgesia

	Duration of surgery		
Drug	Short (1–2 h)	Long (> 2 h)	Notes
Preoperative (day prior)			
Tylenol	975 mg TID	975 mg TID	
Ibuprofen	800 mg TID	800 mg TID	
Holding room			
Gabapentin	600 mg	600 mg	
Celebrex	450 mg	450 mg	
Tylenol	975 mg	975 mg	
Glycopyrolate	0.2–0.3 mg	0.2–0.3 mg	
Intraoperative			
Induction			
Ketamine	Bolus	Bolus and infusion	Bolus 0.25 mg/kg Infusion 4 μg/kg/min
Lidocaine	Bolus	Infusion	Bolus 1 mg/kg Infusion 1 mg/kg/h
Dexmetatomidine		Bolus and infusion	Bolus 1 μg/kg Infusion 0.1 μg/kg/h
Magnesium	Bolus	Bolus and infusion	Bolus 2 g Infusion 1 g/h
Analgesics (provider discretion)			
Toradol	Bolus	Bolus	15–30 mg
Hydromorphone	Bolus	Bolus	0.2–0.4 mg
Morphine	Bolus	Bolus	0.06–0.1 mg/kg
PACU			
Analgesics			
Fentanyl	25 μg/dose	25 μg/dose	
Hydromorphone	0.2–0.4 mg/dose	0.2–0.4 mg/dose	
Morphine	4 mg/dose	4 mg/dose	
Antiemetics			
Zofran	4 mg	4 mg	
Dexamethasone	4–8 mg	4–8 mg	
Haldol	1–2 mg	1–2 mg	
Benadryl	6.25 mg	6.25 mg	

In summary, the available evidence suggests that avoiding opioids during anesthesia is feasible without hemodynamic instability. Further studies are needed that look at long-term outcomes such as total postoperative opioid use, duration of this use, and return to function, as well as the incidence of chronic pain. In the current setting of the opioid crisis, we should lead the charge to change our clinical practice to minimize or eliminate the use of perioperative opioids which can potentially have more far reaching effects than simply length of stay or reduced nausea. The new approach in anesthesia can provide hypnosis with amnesia and muscle relaxation at the moment the anesthesiologist or surgeon requires it, while also maintaining sufficient tissue perfusion and sympathetic stability to protect organs.

## Conclusion

Anesthesiologists are leading experts in pain medicine, and through evidence-based implementations such as ERAS and multimodal anesthesia, the specialty is helping to address the opioid crisis by reducing the amount of opioid used in the perioperative period while still maintaining adequate acute pain control. The time has come to change the foundations of our practice from that of an opioid-based one to that of a multimodal practice (Fig. 2), wherein analgesia is managed with non-opioid-based agents first, then layering on additional layers of analgesics saving opioids as the capstone in analgesic management.

Perioperative physicians and anesthesiologists should continue to pursue evidence-based research to assist with the opioid epidemic from a broad, perioperative population health approach. We share the responsibility with the rest of the medical community to not only decrease the financial burden on society and on hospitals but to assist in solving the epidemic, which has now become the number one accidental cause of death in the USA.

## Abbreviations

ERAS: Enhanced recovery after surgery
ERP: Enhanced recovery pathway
IV: Intravenous
LOS: Length of stay
NMDA: *N*-Methyl-d-aspartate
NSAIDs: Non-steroidal anti-inflammatory drugs
OFA: Opioid-free anesthetic
ORD: Opioid-induced respiratory depression
SD: Standard deviation
SSRI: Selective serotonin reuptake inhibitor
TAP: Transversus abdominis plane
